# Clinical characteristics and prognostic analysis of idiopathic inflammatory myopathy with positive anti‐aminoacyl‐tRNA synthetase antibodies: A single center experience

**DOI:** 10.1002/iid3.1085

**Published:** 2023-11-17

**Authors:** Di Zhang, Huijing Wang, Xinpeng Zhou, Jianguo Yang, Yuan Liu, Wenjing Wang, Ping Jiang, Bing Fan

**Affiliations:** ^1^ Department of Rheumatology Affiliated Hospital of Shandong University of Traditional Chinese Medicine Jinan Shandong China; ^2^ Department of Rheumatology Renji Hospital, School of Medicine, Shanghai Jiaotong University Shanghai China; ^3^ College of Traditional Chinese Medicine Shandong University of Traditional Chinese Medicine Jinan Shandong China

**Keywords:** anti‐aminoacyl‐tRNA synthetase antibodies, anti‐synthetase syndrome, interstitial lung disease, prognostic factor

## Abstract

**Objectives:**

To identify the differences of clinical characteristics, laboratory findings, and the long‐term outcomes in patients with anti‐synthetase syndrome (ASS) of different anti‐aminoacyl‐transfer RNA synthetase antibodies.

**Methods:**

We retrospectively enrolled 119 patients with ASS, and the clinical characteristics and laboratory findings were collected. Additionally, multivariate COX regression analysis was performed to estimate the risk factors of prognosis in patients with ASS.

**Results:**

The frequency of interstitial lung disease (ILD) reached 93.3% in our cohort, of 28 (23.5%) was classified as rapidly progressive (RP)‐ILD. The highest incidence of RP‐ILD was 36.4% in the PL12 group of ASS patients. The ILD group was characterized by an older age, a lower prevalence of V sign, and a higher prevalence of pulmonary symptoms when contrasted with the non‐ILD group. There were statistical differences of clinical significance in arthritis, myositis, mechanic's hands, triad, shawl sign, V sign, and Raynaud's phenomenon among the four subgroups (all *p* < .05). Additionally, the prevalence rates of arthritis, myositis, mechanic's hands, triad, and V sign in the anti‐Jo1 antibody‐positive group were significantly higher than anti‐Jo1 antibody‐negative patients with ASS (all *p* < .05). Multivariate Cox regression analysis showed mechanic's hands (odds ratio [OR] = 6.47, *p* < .001), anti‐nuclear antibodies (ANA) (OR = 2.13, *p* = .026), ILD (OR = 10.50, *p* < .001), and V sign (OR = 0.30, *p* = .007) were independent factors affecting the prognosis of patients with ASS. The incidences of RP‐ILD, arthritis, myositis, triad, mechanic's hands, and shawl sign were more frequent in the anti‐Ro52 antibody‐positive group than the anti‐Ro52 antibody‐negative patients with ASS (all *p* < .05).

**Conclusions:**

Patients with ASS accompanied with ILD are highly prevalent. Mechanic's hands, ANA, and ILD may be a potential biomarker for predicting a poor prognosis in patients with ASS. Additionally, the detection of the anti‐Ro52 antibody provides valuable insights for managing and predicting disease progression and long‐term outcomes.

## Graphical abstract

To determine distinct phenotype groups in patients with idiopathic inflammatory myopathy (IIM), and to identify the differences of clinical characteristics, laboratory findings, and the long‐term outcomes in patients with anti‐synthetase syndrome (ASS) of different anti‐aminoacyl‐tRNA synthetase (ARS) antibodies.

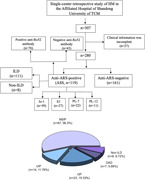

## 1 INTRODUCTION

Idiopathic inflammatory myopathy (IIM) is a group of autoimmune diseases that principally affect the skeleton muscles, but also multiple systems. Anti‐synthetase syndrome (ASS) is characterized by a high serological presence of distinct anti‐aminoacyl‐transfer‐RNA synthetase (ARS) antibodies and is accompanied by clinical manifestations, including arthritis, myositis, fever, Raynaud's phenomenon, and interstitial lung disease (ILD).^1^, ^2^ The adaptive immune mechanisms involved in ASS including antigen‐driven B cell responses in myositis and the presence of clonally expanded T cells in endomysial infiltration. For example, anti‐histidyl (Jo1) antibodies bound to common autoepitopes and altered in titers with disease activity.^3^ Interestingly, CD4^+^ T cells with reactivity against histidyl‐transfer RNA (tRNA) synthetase are found in both the blood and lungs of patients with ASS.^4^ These findings suggest these immune responses were closely associated with muscle and lung damage in patient with ASS.

Although patients with ASS share some common clinical characteristics, multiple studies have revealed the heterogeneity of ASS with different anti‐ARS antibodies.^5^, ^6^, ^7^ The classification of patients with ASS into distinct phenotype subgroups with significant prognostic value has potential to enhance the efficacy of clinical decisions for clinicians.^8^, ^9^

In recent years, due to the advancements and widespread adoption of myositis‐specific autoantibodies (MSAs) and myositis‐associated autoantibodies (MAAs), clinicians experienced a more in‐depth understanding of the diagnosis and treatment management of ASS. For instance, ILD with positive anti‐ARS antibodies progresses rapidly with poor prognosis and low survival rate.^10^, ^11^ However, when individuals present with solitary or non‐specific symptoms, the potential for a high rate of misdiagnosis and missed diagnosis increases, leading to increased costs and delayed treatment. Moreover, there is a scarcity of data regarding the risk factors of patients with IIM and mortality during disease progression and exacerbation, particularly in those with positive anti‐ARS antibodies. Accurate and current data on the prevalence or incidence of ASS remains elusive, largely due to small or non‐representative sample sizes, and incomplete risk factors. Additionally, predictive models tailored to the Chinese population have yet to be established.

In this study, we evaluated disparities in the clinical characteristics, laboratory findings, and long‐term outcomes among ASS patients with various anti‐ARS antibodies. Our aim was to offer valuable evidence for earlier detection, diagnosis, personalized treatment strategies, and prognosis.

## 2 MATERIALS AND METHODS

### 2.1 Population and study design

**Figure 1:**
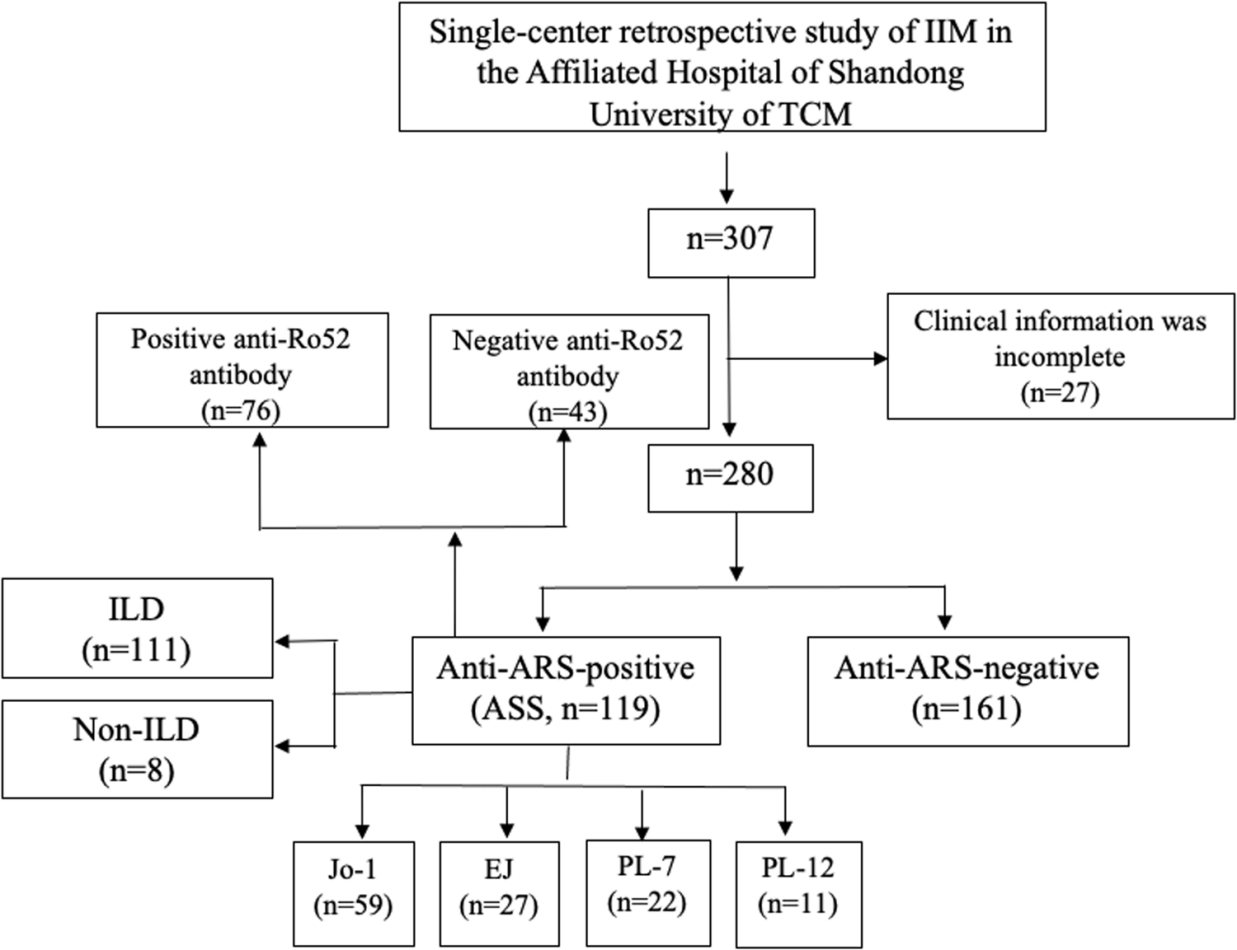
Flow chart of the study. Enrollment and selection of patients. ARS, anti‐aminoacyl‐tRNA synthetase; ASS, anti‐synthetase syndrome; EJ, anti‐glycyl; IIM, idiopathic inflammatory myopathy; Jo1, anti‐histidyl; PL7, anti‐threonyl; PL12, anti‐alanyl; TCM, traditional chinese medicine; tRNA, transfer RNA.

This retrospective study included patients diagnosed with ASS at the Affiliated Hospital of Shandong University of Traditional Chinese Medicine in China from January 2016 to September 2022. The flow chart of inclusion is shown in Figure 1. This study was approved by the Ethical Committee of Shandong University of Traditional Chinese Medicine (2021‐027‐KY).

### 2.2 Data collection

Clinical and laboratory data were obtained from medical records at the time of the initial diagnosis of ASS. Clinical data included age of onset, sex, duration, smoking history, alcohol history, contact history, pulmonary symptoms (cough/sputum/dyspnea), arthritis, myositis, fever, triad, mechanic's hands, shawl sign, V sign, Gottron's papule, heliotrope rash, Raynaud's phenomenon, myalgia, ILD and rapidly progressive (RP)‐ILD. Laboratory data included rheumatoid factor (RF), anti‐cyclic citrullinated peptide antibody (ACPA), erythrocyte sedimentation rate (ESR), C reactive protein (CRP), alanine aminotransferase (ALT), aspartate aminotransferase (AST), creatine kinase (CK), creatine kinase‐MB (CK‐MB), lactate dehydrogenase (LDH), α‐hydroxybutyrate dehydrogenase (αHBDH), ferritin (FER), anti‐nuclear antibody (ANA) and anti‐Ro52 antibody.

### 2.3 Definitions

IIM was defined by the 2017 European League Against Rheumatism/American College of Rheumatology Classification Criteria,^12^ and patients with ASS were diagnosed based on the criteria proposed by Connors et al.^13^ The presence of ILD was evaluated by high‐resolution computed tomography (HRCT) and pulmonary function test. HRCT scan patterns were classified as nonspecific interstitial pneumonia (NSIP), organizing pneumonia (OP), usual interstitial pneumonia (UIP), and diffuse alveolar damage (DAD) by 2 experienced radiologists. RP‐ILD was defined as a worsening of radiologic interstitial changes with progressive dyspnea and hypoxemia within 3 months after the onset of respiratory symptoms.^14^

### 2.4 Anti‐ARS antibodies analysis

The anti‐ARS antibodies included anti‐histidyl (Jo1), anti‐threonyl (PL7), anti‐alanyl (PL12), and anti‐glycyl (EJ) antibodies. All the detections were carried out by immunoblotting technique in Jiangsu Simcere Diagnostic Laboratory (Jiangsu Simcere Diagnostics Co, Ltd.).

### 2.5 Statistical analysis

All statistical analysis was performed using SPSS software version 25.0 (IBM). Continuous variables were described as the median (interquartile range [IQR]). Categorical variables were presented as numbers (percentages). Normally distributed variables between groups were analyzed by the Student *t* test. The Mann–Whitney *U* test was used for those with non‐normal distribution. The Chi‐square test or Fisher's exact test was used to in categorical variables, as required. Cox regression analysis was used to identify independent risk factors, variables selected by univariate Cox regression analysis (*p* < .05) were included in a multivariate Cox regression analysis. The predictive factors were quantified by the odds ratio (OR) with 95% confidence interval. Results were regarded as statistically significant when *p* values were <.05.

## 3 RESULTS

### 3.1 Clinical characteristics and laboratory findings in patients with ASS

A total of 119 diagnosed with ASS were included in this study, and clinical characteristics were listed in Table 1. The median age of onset was 54.0 (IQR: 50.0–60.0), and 88 (73.9%) patients were women. The median disease duration was 24.0 (IQR: 6.0–48.0) months, and 12 (10.1%) patients had a history of smoking. As the most common manifestation of ASS, the frequencies of pulmonary symptoms (cough/sputum/dyspnea) and ILD both reached 93.3% in our study, of 28 (23.5%) were classified as RP‐ILD. Among patients with ASS, 67 (56.3%) exhibited Mechanic's hands, while 59 (49.6%) had arthritis, 53 (44.5%) experienced myositis, 20 (16.8%) presented with Gottron papules, 18 (15.1%) had Raynaud's phenomenon, 17 (14.3%) displayed heliotrope rash, 35 (29.4%) had triad, 13 (10.9%) experienced fever, 10 (8.4%) had V sign, and 7 (5.9%) showed shawl sign. The median of serum CK, ESR, and FER levels were 141.0 (IQR: 42.0–454.5), 27.0 (IQR: 15.5–48.5) and 181.4 (IQR: 112.5–364.1), respectively. The frequencies of ANA and anti‐Ro52 antibodies in patients with ASS were notably high, with the rate of 79.0% and 63.9%, respectively.

**Table 1:** Clinical characteristics and laboratory findings between ILD and non‐ILD groups in patients with anti‐synthetase syndrome (ASS).

Variables	ASS (*n* = 119)	ILD (*n* = 111)	Non‐ILD (*n* = 8)	*p* value^a^
Female, *n* (%)	88 (73.9)	81 (73)	7 (87.5)	0.679
Age of onset, median (IQR), years	54.0 (50.0, 60.0)	54.0 (50.0, 60.0)	46.5 (35.5, 52.5)	0.034
Duration, median (IQR), months	24.0 (6.0, 48.0)	24.0 (6.0, 48.0)	6.0 (2.5, 24.0)	0.146
Smoking history, *n* (%)	12 (10.1)	12 (10.8)	0 (0)	1.000
Alcohol history, *n* (%)	5 (4.2)	5 (4.5)	0 (0)	1.000
Contact history, *n* (%)	6 (5)	6 (5.4)	0 (0)	1.000
ILD, *n* (%)	111 (93.3)			
RP‐ILD, *n* (%)	28 (23.5)	25 (22.5)	0 (0)	0.201
Pulmonary symptoms (cough/sputum/dyspnea), n (%)	111 (93.3)	107 (96.4)	4 (50.0)	<0.001
Arthritis, *n* (%)	59 (49.6)	53 (47.7)	6 (75.0)	0.163
Myositis, *n* (%)	53 (44.5)	51 (45.9)	2 (25.0)	0.296
Fever, *n* (%)	13 (10.9)	12 (10.8)	1 (12.5)	1.000
Triad, *n* (%)	35 (29.4)	35 (31.5)	0 (0)	0.103
Mechanic's hands, *n* (%)	67 (56.3)	63 (56.8)	4 (50.0)	0.728
Shawl sign, *n* (%)	7 (5.9)	7 (6.3)	0 (0)	1.000
V sign, *n* (%)	10 (8.4)	7 (6.3)	3 (37.5)	0.019
Gottron's papule, *n* (%)	20 (16.8)	19 (17.1)	1 (12.5)	1.000
Heliotrope rash, *n* (%)	17 (14.3)	14 (12.6)	3 (37.5)	0.087
Raynaud's phenomenon, *n* (%)	18 (15.1)	15 (13.5)	3 (37.5)	0.100
RF, median (IQR), IU/mL	10.1 (10.1, 11.2)	10.1 (10.1, 11.2)	10.1 (9.0, 14.3)	0.513
ACPA, median (IQR), RU/mL	25.0 (25.0, 25.0)	25.0 (25.0, 25.0)	25.0 (25.0, 25.0)	0.960
ESR, median (IQR), mm/h	27.0 (15.5, 48.5)	26.0 (15.0,48.5)	35.5 (21.0, 62.0)	0.474
CRP, median (IQR), mg/L	7.7 (3.3, 33.8)	6.8 (3.3, 32.5)	11.0 (5.6, 66.4)	0.265
ALT, median (IQR), units/L	24.0 (17.0, 41.0)	24.0 (17.0, 41.0)	25.5 (20.5, 35.0)	0.915
AST, median (IQR), units/L	24.0 (19.0, 38.0)	24.0 (19.0,37.0)	27.5 (17.5, 52.5)	0.937
CK, median (IQR), units/L	141.0 (42.0, 454.5)	143.0 (42.0, 452.0)	67.5 (36.0, 590.0)	0.656
CK‐MB, median (IQR), ng/mL	12.8 (7.5, 23.8)	13.0 (8.5, 24.0)	8.0 (4.9, 13.6)	0.208
LDH, median (IQR), units/L	257.0 (193.0, 321.5)	257.0 (194.5, 321.5)	226.0 (148.5, 337.0)	0.402
αHBDH, median (IQR), units/L	174.0 (132.5, 235.5)	174.0 (134.0, 235.5)	142.0 (101.5, 230.5)	0.301
FER, median (IQR), ng/mL	181.4 (112.5, 364.1)	181.4 (107, 1364.1)	209.8 (127.7, 336.4)	0.629
ANA, *n* (%)	94 (79)	88 (79.3)	6 (75.0)	0.674
Anti‐Ro52 antibody, *n* (%)	76 (63.9)	72 (64.9)	4 (50.0)	0.458

Abbreviations: ACPA, anti‐citrullinated protein autoantibodies; ALT, alanine aminotransferase; ANA, anti‐nuclear antibodies; ASS, anti‐synthetase syndrome; AST, aspartate aminotransferase; CK, creatine kinase; CK‐MB, creatine kinase‐myocardial band; CRP, C‐reactive protein; ESR, erythrocyte sedimentation rate; FER, ferroprotein; αHBDH, α‐hydroxybutyrate dehydrogenase; ILD, interstitial lung disease; IQR, interquartile range; LDH, lactic dehydrogenase; RF, rheumatoid factor; RP‐ILD, rapidly progressive interstitial lung disease.

^a^ *p* between ILD and non‐ILD groups.

### 3.2 Comparisons of clinical characteristics and laboratory findings between ILD and non‐ILD groups in patients with ASS

As shown in Table 1, the ASS patients in the ILD group were characterized by an older age, a lower prevalence of V sign, and a higher prevalence of pulmonary symptoms when contrasted with the non‐ILD group (*p* = .034, *p* < .001, and *p* = .019, respectively). But no statistical differences were observed in other clinical characteristics and laboratory findings between the two groups, such as the prevalence of ANA, anti‐Ro52 antibody, and the median of serum RF, ACPA, ESR, CRP, ALT, AST, CK, CK‐MB, LDH, αHBDH, and FER levels.

### 3.3 Comparisons of clinical characteristics and laboratory findings among four subgroups in patients with ASS

**Figure 2:**
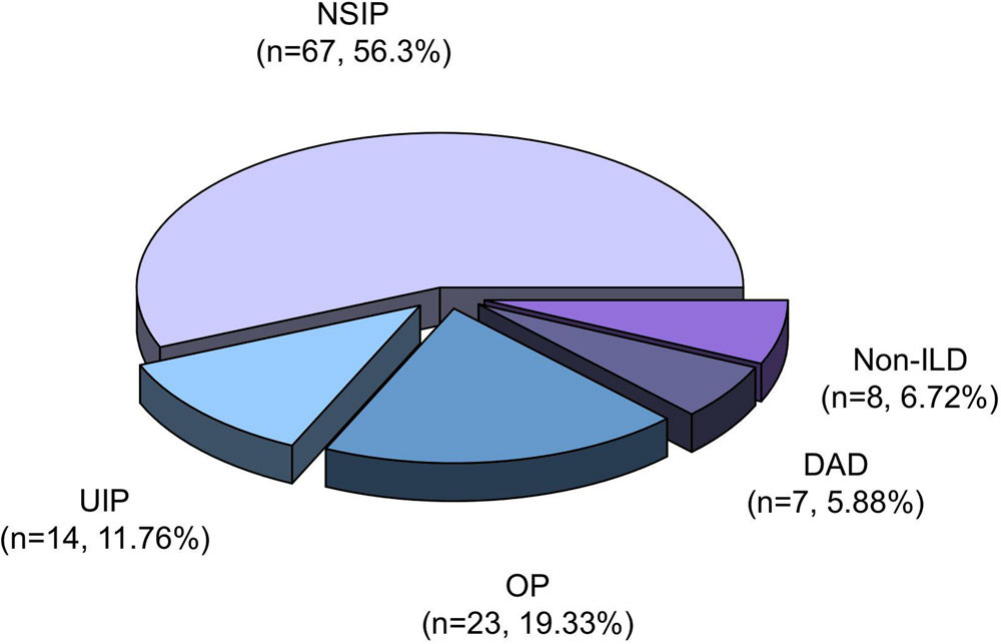
Distribution of interstitial lung disease (ILD) in patients with anti‐synthetase syndrome (ASS). DAD, diffuse alveolar damage; NSIP, nonspecific interstitial pneumonia; OP, organizing pneumonia; UIP, usual interstitial pneumonia.

Among 119 patients with ASS, 59 patients (49.6%) were anti‐Jo1‐positive, 27 (22.7%) were anti‐EJ‐positive, 22 (18.5%) were anti‐PL7‐positive, and 11 (9.2%) were anti‐PL12‐positive (Table 2). No statistical differences were observed in the demographic features (female, age, and duration), smoking history, alcohol history and contact history among the four subgroups of patients with ASS. All the overall prevalence of ILD across all four subgroups exceeded 90.0%, even reaching 100% in patients with the anti‐PL12 antibody, and the highest incidence of RP‐ILD was also in the PL12 group (36.4%), followed by the PL7 group (31.8%), EJ group (22.2%), Jo1 group (13.6%). Based on the patterns observed on HRCT scans, ILD patients were divided into four groups: NSIP (*n* = 67), OP (*n* = 23), UIP (*n* = 14), and DAD (*n* = 7). The distribution of ILD in all patients with ASS was shown in Figure 2. Notably, the most prevalent HRCT pattern of ILD among patients with ASS was NSIP (60.3%), followed by OP (20.7%), UIP (12.6%), and DAD (6.3%). There were statistical differences of clinical manifestations in arthritis, myositis, mechanic's hands, triad, shawl sign, V sign, and Raynaud's phenomenon among the four subgroups (all *p* < .05), but no significant statistical difference was observed in other clinical characteristics (ILD, RP‐ILD, pulmonary symptoms, fever, Gottron's papule, and heliotrope rash) among the four subgroups. Intriguingly, the prevalence rates of arthritis, myositis, mechanic's hands, triad, and V sign in the anti‐Jo1 antibody‐positive group were significantly higher than anti‐Jo1 antibody‐negative (non‐Jo1) patients with ASS (all *p* < .05). But there were no significant differences in the occurrence of ILD and RP‐ILD in relation to the presence of anti‐Jo1 antibody.

**Table 2:** Comparisons of clinical characteristics and laboratory findings among four subgroups in patients with ASS.

Variables	Jo1 (*n* = 59)	EJ (*n* = 27)	PL7 (*n* = 22)	PL12 (*n* = 11)	*p* value^a^	*p* value^b^
Female, *n* (%)	44 (74.6)	20 (74.1)	16 (72.7)	8 (72.7)	1.000	1.000
Age of onset, median (IQR), years	53.0 (49.5, 59.0)	57.0 (50.0, 62.0)	54.5 (51.0, 60.0)	53.0 (45.5, 63.5)	0.730	0.336
Duration, median (IQR), months	12.0 (3.0, 48.0)	24.0 (12.0, 66.0)	18.0 (8.0, 36.0)	12.0 (3.0, 36.0)	0.198	0.149
Smoking history, *n* (%)	5 (8.5)	3 (11.1)	3 (13.6)	1 (9.1)	0.904	0.784
Alcohol history, *n* (%)	1 (1.7)	3 (11.1)	1 (4.5)	0 (0)	0.163	0.364
Contact history, *n* (%)	2 (3.4)	3 (11.1)	1 (4.5)	0 (0)	0.391	0.679
ILD, *n* (%)	54 (91.5)	26 (96.3)	20 (90.9)	11 (100.0)	0.813	0.491
NSIP, *n*	33	14	13	7		
UIP, *n*	5	7	2	0		
OP, *n*	12	3	5	3		
DAD, *n*	4	2	0	1		
RP‐ILD, *n* (%)	8 (13.6)	6 (22.2)	7 (31.8)	4 (36.4)	0.131	0.080
Pulmonary symptoms (cough/sputum/dyspnea), *n* (%)	53 (89.8)	27 (100.0)	20 (90.9)	11 (100.0)	0.289	0.163
Arthritis, *n* (%)	45 (76.3)	8 (29.6)	4 (18.2)	2 (18.2)	<0.001	<0.001
Myositis, *n* (%)	40 (67.8)	7 (25.9)	3 (13.6)	3 (27.3)	<0.001	<0.001
Fever, *n* (%)	7 (11.9)	4 (14.8)	0 (0)	2 (18.2)	0.184	0.974
Triad, *n* (%)	29 (49.2)	5 (18.5)	0 (0)	1 (9.1)	<0.001	<0.001
Mechanic's hands, *n* (%)	47 (79.7)	9 (33.3)	5 (22.7)	6 (54.5)	<0.001	<0.001
Shawl sign, *n* (%)	1 (1.7)	4 (14.8)	0 (0)	2 (18.2)	0.013	0.114
V sign, *n* (%)	1 (1.7)	4 (14.8)	3 (13.6)	2 (18.2)	0.021	0.017
Gottron's papule, *n* (%)	11 (18.6)	2 (7.4)	3 (13.6)	4 (36.4)	0.165	0.775
Heliotrope rash, *n* (%)	5 (8.5)	3 (11.1)	5 (22.7)	4 (36.4)	0.056	0.125
Raynaud's phenomenon, *n* (%)	5 (8.5)	4 (14.8)	8 (36.4)	1 (9.1)	0.026	0.08
RF, median (IQR), IU/mL	10.1 (10.1, 11.0)	10.1 (9.2, 11.3)	10.1 (9.4, 11.2)	10.6 (10.1, 11.2)	0.316	0.169
ACPA, median (IQR), RU/mL	25.0 (25.0, 25.0)	25.0 (25.0, 25.0)	25.0 (25.0, 25.0)	25.0 (25.0, 25.0)	0.616	0.271
ESR, median (IQR), mm/h	25.0 (12.5, 39.0)	28.0 (16.5, 59.0)	33.5 (18.0, 72.0)	45.0 (21.5, 51.0)	0.191	0.032
CRP, median (IQR), mg/L	4.7 (3.1, 17.3)	10.3 (5.0, 30.0)	12.1 (3.3, 51.6)	18.4 (5.6, 37.7)	0.126	0.025
ALT, median (IQR), units/L	28.0 (18.0, 41.1)	23.0 (18.5, 39.0)	21.0 (13.0, 32.0)	19.0 (15.0, 34.0)	0.142	0.056
AST, median (IQR), units/L	30.0 (20.5, 43.0)	24.0 (19.5, 39.3)	20.5 (19.0, 33.0)	23.0 (18.0, 28.0)	0.285	0.098
CK, median (IQR), units/L	206.0 (68.5, 542.5)	158.0 (60.5, 780.0)	77.5 (32.0, 163.0)	30.0 (25.0, 40.0)	0.002	0.022
CK‐MB, median (IQR), ng/mL	14.0 (8.0, 24.0)	13.0 (9.6, 30.5)	11.0 (8.0, 15.0)	9.0 (2.2, 10.1)	0.066	0.311
LDH, median (IQR), units/L	262.0 (192.0, 323.0)	275.0 (207.5, 382.5)	250.0 (193.0, 290.0)	200.0 (173.0, 252.5)	0.323	0.975
αHBDH, median (IQR), units/L	174.0 (124.5, 240.5)	185.0 (151.0, 257.0)	164.5 (138.0, 197.0)	139.0 (133.5, 179.5)	0.277	0.748
FER, median (IQR), ng/mL	138.8 (101.0, 280.8)	173.4 (128.2, 475.3)	205.2 (121.2, 381.9)	321.9 (191.5, 728.0)	0.415	0.151
ANA, *n* (%)	43 (72.9)	23 (85.2)	19 (86.4)	9 (81.8)	0.493	0.162
Anti‐Ro52 antibody, *n* (%)	39 (66.1)	15 (55.6)	13 (59.1)	9 (81.8)	0.444	0.755

Abbreviations: ASS, anti‐synthetase syndrome; DAD, diffuse alveolar damage; EJ, anti‐glycyl; IQR, interquartile range; Jo1, anti‐histidyl; NSIP, antibodies nonspecific interstitial pneumonia; OP, organizing pneumonia; PL7, anti‐threonyl; PL12, anti‐alanyl; UIP, usual interstitial pneumonia.

^a^ *p* among the four subgroups of patients with ASS.

^b^ *p* between Jo1 and non‐Jo1 groups.

The median of serum CK levels were ranked highest in the Jo1 group, followed by the EJ group, the PL7 group, and the PL12 group. There was a significantly statistical difference in CK levels among the 4 subgroups of patients with ASS (*p* < .05). However, there were no statistically significant clinical differences in RF, ACPA, ESR, CRP, ALT, AST, CK‐MB, LDH, αHBDH, FER, and the prevalence of ANA and anti‐Ro52 antibodies among all four subgroups. The median of serum ESR, CRP, and CK levels in the Jo1 group were significantly higher than the non‐Jo1 group (*p* < .05).

### 3.4 Prognostic factors in patients with ASS

To identify risk factor of long‐term prognosis in patients with ASS, the multivariate Cox regression analysis was performed. The results of the multivariate analysis showed mechanic's hands (OR = 6.47, 3.05–13.72, *p* < .001), ANA (OR = 2.13, 1.09–4.16, *p* = .026), ILD (OR = 10.50, 4.36–25.24, *p* < .001), and V sign (OR = 0.30, 0.12–0.72, *p* = .007) were independent factors affecting the prognosis of patients with ASS (Table 3).

**Table 3:** Multivariate Cox regression analysis of prognostic factors in patients with ASS.

Variables	OR	(95% CI)	*p* value
Mechanic's hands	6.47	(3.05, 13.72)	<.001
V sign	0.30	(0.12, 0.72)	.007
ANA	2.13	(1.09, 4.16)	.026
ILD	10.50	(4.36, 25.24)	<.001

Abbreviations: ASS, anti‐synthetase syndrome; CI, confidence interval; OR, odds ratio.

### 3.5 Comparisons of clinical characteristics and laboratory findings between positive and negative anti‐Ro52 antibody groups in patients with ASS

The incidences of RP‐ILD, arthritis, myositis, triad, mechanic's hands, and shawl sign were more frequent in the anti‐Ro52 antibody‐positive group than the anti‐Ro52 antibody‐negative patients with ASS (*p* = .0.034, = 0.009, <.001, <.001, *=* 0.010, and = 0.048, respectively). Among patients with ASS, the median of serum AST level in the anti‐Ro52 antibody‐positive group was significantly higher than the anti‐Ro52 antibody‐negative group (*p* < .05). No significant differences were observed in other clinical characteristics and laboratory findings (Table S1).

## 4 DISCUSSION

In our study, we estimate the differences between patients with ASS with various anti‐ARS antibodies in clinical characteristics, laboratory findings and prognostic factors. Further, comparisons of clinical and serological outcomes between ILD and non‐ILD groups, positive and negative anti‐Ro52 antibody groups were performed in patients with ASS.

The frequent presence of auto‐immune antibodies in IIM underlines the significant role of the immune system in the pathophysiology. The immune cells phenotypes and inflammatory products in the organs most frequently affected, including muscle, skin and the lungs, have been reported in the newly identified subgroups of IIM defined by ASS.^15^ The anti‐Ro52 antibody, recognized as one of the myositis‐related antibodies, was found in 76 out of 119 patients with ASS (63.9%) in our study. This antibody not only commonly serves more frequently served as a serological marker for various autoimmune diseases, including Sjogren syndrome, rheumatoid arthritis, and systemic lupus erythematosus but also detected in patients with IIM.^16^, ^17^, ^18^ Conversely, MSAs were specific to IIM. However, many individuals who lack typical characteristics, such as rash, arthritis, and myositis, often went unrecognized due to the absence of anti‐ARS antibody testing. Moreover, a poor standardization of ANA assays, particularly for MSAs, can lead to negative ANA results potentially diverting clinicians away from autoimmune disease diagnoses. This delay in diagnosis and treatment can worsen the prognosis.^19^ Indeed, a negative ANA test result does not exclude the possible diagnosis of ASS or inflammatory myopathies.^20^

ASS patients with ILD were frequently misdiagnosed with idiopathic interstitial pneumonia during their first visit to the respiratory department, which required a comprehensive assessment by multidisciplinary experts (rheumatologists, pulmonologists, and radiologists).^21^ The presence of ILD typically contributed to increased mortality rates in patients with ASS.^22^ In our study, results from multivariate Cox regression analysis confirmed that ILD stood as an independent risk factor for poor prognosis in patients with ASS. The overall prevalence of ILD across all four subgroups exceeded 90.0% and even reached 100% in patients with the anti‐PL12 antibody, who exhibited the highest incidence of RP‐ILD. Additionally, patients in the ILD group tended to be older, and displayed a higher proportion of pulmonary symptoms (cough/sputum/dyspnea) compared to the non‐ILD group. The occurrence of RP‐ILD was more frequently observed in the positive anti‐Ro52 antibody group than those with negative anti‐Ro52 antibody among patients with ASS. These findings were consistent with previous reports.^23^, ^24^, ^25^, ^26^, ^27^, ^28^

It has been stated the heterogeneity existed among distinct anti‐ARS antibodies.^29^ Both anti‐Ro52 antibodies and any anti‐ARS antibodies were pertinent to clinical outcomes. Specifically, anti‐PL12, and anti‐Ro52 antibodies were closely associated with a higher prevalence of ILD and more severe lung involvement. Individuals testing positive for anti‐ARS antibodies, especially when combined with anti‐Ro52 antibodies, tend to experience the most unfavorable prognosis.^30^, ^31^, ^32^, ^33^ Old age and RP‐ILD have been identified as predictors of poor prognosis in patients with ASS.^34^ Therefore, the occurrence of ILD was significantly associated with poor outcomes in patients with ASS. The relationship between anti‐Ro52 antibody and ASS was required to further exploration. Similar to one previous result,^35^ the V sign was an independent protective factor for good prognosis in patients with ASS.

Anti‐Jo1 antibody is among the most frequently detected MSAs.^36^ Previous research has highlighted that patients with positive anti‐Jo1 antibody often presented incomplete clinical characteristics. They may present with joint, fever of unknown origin, pulmonary or muscle involvement, which can lead to prolonged misclassification as conditions such as arthritis or pneumonia before receiving a definitive diagnosis of ASS.^37^ Furthermore, ACPA has been established as a serological marker for erosive arthritis in anti‐Jo1‐antibody‐positive patients with ASS.^38^ The results of this study demonstrated that individuals in the positive anti‐Jo1 antibody group were more frequently relevant to arthritis, myositis, mechanic's hands, triad, and V sign in contrast to the negative anti‐Jo1 antibody group among patients with ASS. The median of serum ESR, CRP, and CK levels were higher in the positive anti‐Jo1 antibody group than the non‐Jo1 group. It has been reported that anti‐Jo1 antibody directed against histidyl‐tRNA synthetase can emerge months before clinical manifestations. This is accompanied by spectrotype broadening, class switching, and an experience affinity maturation to that antigen,^39^ which partly explains why patients with positive anti‐Jo1 antibodies are more likely to present with the classic triad of myositis.

Complexity arises from the variability in presentation and disease courses, as well as the multiorgan and systemic characteristics of ASS.^15^ It's worth noting that the discovery that mice, when immunized with histidyl‐tRNA synthetase, develop anti‐synthetase autoantibodies and exhibit muscle and lung infiltration, provides evidence that the immune response in ASS may target endothelial cells, muscle cells, and lung tissue.^40^ To date, numerous prognostic biomarkers associated with ASS have been reported. More favorable prognostic factors for outcomes in ASS patients could help physicians and patients improved treatment and management of this disease. Thus, it's essential to focus on recognizing and distinguishing the similarities and differences among ASS with distinct anti‐ARS antibodies. This approach will allow us to better understand the rapidly progressing features and actively seek subtle clues for improved disease management. An earlier, accurate diagnosis and promptly efficient treatment benefited patient survival.

There are several constraints in the present study. First, this is a retrospective, single‐center study, which lead to information bias. Second, the sample size is somewhat small. Third, the follow‐up durations vary, and a longer follow‐up time might yield different outcomes. Although we took to minimize information bias by data collection standardization, blinding and objectivity, quality control measures, a multicenter and larger population‐size study with multiple comparison will be needed to overcome these limitations and validate these results.

## 5 CONCLUSION

In this study, patients with ASS accompanied with ILD are highly prevalent. Importantly, older age, a higher proportion of pulmonary symptoms, and a lower proportion of V sign were significantly associated with ILD. Mechanic's hands, ANA, and ILD may be a potential biomarker for predicting a poor prognosis in patients with ASS. Additionally, the detection of anti‐Ro52 antibody can help clinical physicians predict disease progression and long‐term prognosis for the management of patients with ASS.

## AUTHOR CONTRIBUTIONS

**Di Zhang**: conceptualization; data curation; investigation; validation; visualization; writing—original draft; writing—review & editing. **Huijing Wang**: conceptualization; investigation; validation; writing—original draft; writing—review & editing. **Xinpeng Zhou**: conceptualization; data curation; investigation; writing—original draft; Jianguo Yang: Data curation; investigation; visualization. **Yuan Liu**: data curation; investigation. **Wenjing Wang**: data curation; investigation; Ping Jiang: Fungding acquisition; supervision. **Bing Fan**: conceptualization; investigation; writing—original draft; writing –review & editing.

## CONFLICT OF INTEREST STATEMENT

The authors declare no conflicts of interest.

## ETHICS STATEMENT

This study was approved by the Ethical Committee of Shandong University of Traditional Chinese Medicine (2021‐027‐KY).

## Supporting information

Supporting information.

## Data Availability

The data used to support the findings of this study are available from the corresponding author upon request. All data related to this article are available from the corresponding author upon reasonable request.
