# Supplementary material for: Clinical characteristics and prognostic analysis of idiopathic inflammatory myopathy with positive anti‐aminoacyl‐tRNA synthetase antibodies: A single center experience
Source: Immun Inflamm Dis. 2023 Nov 17;11(11):e1085. doi: 10.1002/iid3.1085 (PMC10655634; doi:10.1002/iid3.1085)
Supplement: Supplementary file 1 — Supporting information. [file IID3-11-e1085-s001.docx]

**Supplementary Table 1.** Comparisons of clinical characteristics and laboratory findings between positive anti-Ro52 antibody and negative anti-Ro52 antibody groups in patients with antisynthetase syndrome (ASS).

| Variables | Positive anti-Ro52 antibody (n = 76) | Negative anti-Ro52 antibody (n = 43) | *P-*value |
| --- | --- | --- | --- |
| Female, n (%) | 60 (78.9) | 28 (65.1) | 0.152 |
| Age of onset, median (range), years | 54.0 (50.0, 60.0) | 53.0 (48.5, 60.5) | 0.652 |
| Duration, median (range), months | 19.0 (6.5, 48.0) | 24.0 (3.0, 42.0) | 0.370 |
| Smoking history, n (%) | 7 (9.2) | 5 (11.6) | 0.755 |
| Alcohol history, n (%) | 3 (3.9) | 2 (4.7) | 1.000 |
| Contact history, n (%) | 4 (5.3) | 2 (4.7) | 1.000 |
| ILD, n (%) | 72 (94.7) | 39 (90.7) | 0.458 |
| RP-ILD, n (%) | 21 (27.6) | 4 (9.3) | 0.034 |
| Pulmonary symptoms  (cough/sputum/dyspnea),  n (%) | 71 (93.4) | 40 (93.0) | 1.000 |
| Arthritis, n (%) | 45 (59.2) | 14 (32.6) | 0.009 |
| Myositis, n (%) | 45 (59.2) | 8 (18.6) | <0.001 |
| Fever, n (%) | 8 (10.5) | 5 (11.6) | 1.000 |
| Triad, n (%) | 33 (43.4) | 2 (4.7) | <0.001 |
| Mechanic's hands, n (%) | 50 (65.8) | 17 (39.5) | 0.010 |
| Shawl sign, n (%) | 7 (9.2) | 0 (0) | 0.048 |
| V sign, n (%) | 9 (11.8) | 1 (2.3) | 0.092 |
| Gottron's papule, n (%) | 14 (18.4) | 6 (14.0) | 0.711 |
| Heliotrope rash, n (%) | 11 (14.5) | 6 (14.0) | 1.000 |
| Raynaud's phenomenon, n (%) | 9 (11.8) | 9 (20.9) | 0.288 |
| RF, median (range), IU/ml | 10.1 (9.4, 11.2) | 10.1 (10.1, 11.2) | 0.587 |
| ACPA, median (range), RU/ml | 25.0 (25.0, 25.0) | 25.0 (25.0, 25.0) | 0.265 |
| ESR, median (range), mm/h | 26.0 (16.5, 52.0) | 30.0 (15.0, 44.0) | 0.993 |
| CRP, median (range), mg/l | 8.4 (3.3, 36.5) | 5.8 (3.1, 24.8) | 0.183 |
| ALT, median (range), units/l | 26.0 (17.0,50.0) | 23.0 (16.5,31.9) | 0.144 |
| AST, median (range), units/l | 29.0 (19.5, 47.5) | 22.0 (18.5, 31.5) | 0.036 |
| CK, median (range), units/l | 158.0 (41.5, 590.0) | 85.0 (47.5, 334.0) | 0.293 |
| CK-MB, median (range), ng/ml | 12.9 (7.0, 24.0) | 12.0 (8.0, 16.5) | 0.890 |
| LDH, median (range), units/l | 270.5 (195.5, 331.5) | 240.0 (192.5, 303.0) | 0.243 |
| αHBDH, median (range), units/l | 178.0 (132.5, 244.0) | 159.0 (132.5, 199.0) | 0.273 |
| FER, median (range), ng/ml | 177.4 (107.1, 364.1) | 188.9 (119.2, 357.0) | 0.769 |
| ANA, n (%) | 60 (78.9) | 34 (79.1) | 1.000 |

**Abbreviations:** IQR, interquartile range; ILD, interstitial lung disease; RP-ILD, rapidly progressive interstitial lung disease; RF, rheumatoid factor; ACPA, anti-citrullinated protein autoantibodies; ESR, erythrocyte sedimentation rate; CRP, C-reactive protein; ALT, alanine aminotransferase; AST, aspartate aminotransferase; CK, creatine kinase; CK-MB, creatine kinase-myocardial band; LDH, lactic dehydrogenase; αHBDH, α-hydroxybutyrate dehydrogenase; FER, ferroprotein; ANA, antinuclear antibodies.
